# Best evidence summary for self-management of radiodermatitis in head and neck cancer patients: an integrative review

**DOI:** 10.3389/fonc.2025.1693170

**Published:** 2025-11-13

**Authors:** Lianfang Cheng, Mingqi Zhang, Yicen Zheng, Liyu Lin, Tingting Xiao, Man Zhang, Meiling Huang, Lichun Xu

**Affiliations:** 1 Department of Nursing, Zhongshan Hospital Xiamen University, Xiamen, China; 2 School of Nursing, Fujian University of Traditional Chinese Medicine, Fuzhou, China; 3 Nursing College, Fujian Medical University, Fuzhou, China; 4 Xiamen Nursing Quality Control Center, Xiamen, China

**Keywords:** head and neck cancer, radiation dermatitis, self-management, evidence summary, evidence-based nursing

## Abstract

**Aim:**

To evaluate and synthesize the best evidence for radiodermatitis self-management in head and neck cancer (HNC) patients, and to provide evidence-based guidance for improving their self-management capabilities.

**Design:**

integrative review.

**Data Sources:**

We conducted a comprehensive search across integrated English and Chinese databases, relevant guideline networks, and official association websites. The search period was from January 1, 2000, to August 31, 2024, identifying literature on self-managing radiotherapy-induced dermatitis in HNC patients.

**Review Methods:**

Studies were screened for eligibility based on the inclusion criteria, such as relevance to the self-management of radiation dermatitis in HNC patients.The methodological quality of the included studies was then independently assessed by two reviewers using established appraisal tools specific to each study design to ensure the inclusion of high-quality evidence.

**Results:**

A total of 21 documents were ultimately included, comprising 7 guidelines, 6 expert consensuses, 2 evidence summaries, 2 recommended practices, 2 clinical decisions, and 2 systematic reviews. These sources yielded 56 pieces of evidence, summarized across six key aspects: learning related knowledge, daily life management, self-monitoring, symptom management, dietary management, and psychosocial support.

**Conclusion:**

This study provides a comprehensive summary of the best evidence for radiodermatitis self-management in HNC patients. This compilation can serve as a valuable reference for healthcare professionals guiding patient self-management, thereby facilitating a more scientific and effective approach to patient self-care.

**Impact:**

This review underscores that promoting self-management of radiation dermatitis in HNC patients is crucial for fostering active patient participation in recovery, which may ultimately enhance adherence and improve long-term outcomes.

## 1 Introduction

Head and neck cancer (HNC) typically refers to a group of malignant tumors that originate from soft tissues such as the oral cavity (including the lips), nasal cavity, paranasal sinuses, pharynx, larynx, and salivary glands (1). Recent global data from 2022 indicate nearly 950,000 new HNC cases and approximately 480,000 deaths annually, reflecting a steady increase and underscoring its escalating global public health burden (2). Currently, radiotherapy remains a cornerstone of comprehensive HNC treatment (3, 4). However, despite continuous advancements in modern radiotherapy techniques, unavoidable damage to surrounding normal tissues often leads to varying degrees of acute and chronic complications, with radiation dermatitis being the most common and earliest manifestation (5, 6). The delicate nature of neck skin, coupled with its rich sweat and sebaceous glands and susceptibility to friction, renders this area more vulnerable to radiation dermatitis even at equivalent radiation doses (7). Radiation dermatitis,defined as an inflammatory skin and mucosal lesion caused by various types of ionizing radiation (e.g., beta rays, gamma rays, X-rays, proton beams, and other high-energy particle rays) (8), profoundly impacts HNC patients across physiological, psychological, and social dimensions. Acute radiation dermatitis manifests as hyperpigmentation, pruritus, erythema, and desquamation (9), and without timely intervention, can lead to severe consequences such as intense pain, necrosis, hemorrhage, and infection (8). Conversely, chronic radiation dermatitis is primarily characterized by telangiectasia, skin fibrosis, edema, and ulceration (10), with skin fibrosis potentially even inducing symptoms like dysphagia and aspiration (11, 12). Furthermore, radiotherapy-induced skin damage can result in head and neck deformities, scarring, edema, and skin hyperpigmentation, leading to significant cosmetic alterations (13). Some studies indicate that radiation dermatitis can emerge even years after radiotherapy completion and, once established, is often irreversible. The lack of universally accepted prevention and management standards, both domestically and internationally, underscores the necessity for patients to engage in long-term self-preventive measures (14).

As medical technology advances, outpatient radiotherapy is becoming an increasingly favored option for cancer patients offering treatment quality comparable to inpatient care (15). This has resulted in shorter hospital stays for HNC patients, consequently elevating their need for outpatient care. However, insufficient health education may hinder patients from promptly recognizing early dermatitis symptoms, leading to reactive coping strategies and delayed treatment. Consequently, the early identification of radiation dermatitis and effective guidance for patient self-management prove crucial for ensuring treatment efficacy and enhancing patient quality of life. Cancer patient self-management encompasses behaviors developed by patients during diagnosis and treatment to manage symptoms, psychosocial aspects, treatment regimens, and daily living (16). Within oncology, the clinical value of self-management is well-recognized, with a growing body of related research. However, findings from several recent status surveys (17–19) indicate that most HNC patients exhibit suboptimal self-management capabilities. This deficiency may stem from patients’ insufficient knowledge and skills for self-management, a lack of symptom recognition and coping abilities, and insufficient emotional regulation, all of which significantly impede the recovery. Given these challenges, enhancing HNC patients’ self-management capabilities for radiation dermatitis is particularly urgent and vital. In recent years, while the number of guidelines, evidence summaries, and expert consensuses concerning radiation dermatitis has increased (8, 14), these resources are primarily tailored for clinical healthcare professionals and radiation technologists. Evidence specifically addressing patient self-management remains fragmented, making it difficult for patients and their families to readily access and apply this information in their daily lives. Therefore, this study aims to evaluate and synthesize existing research evidence related to self-management of radiation dermatitis in HNC patients, in order to construct a systematic and targeted best evidence summary that can provide an evidence-based foundation for clinical nursing practice.

## 2 Methods

### 2.1 Evidence retrieval

Following the ‘6S’ pyramid model of evidence-based medicine, searches were conducted from top to bottom (20).This involved searching guideline databases and official websites (including BMJ Best Practice, UpToDate, JBI Evidence Synthesis, Guidelines International Network (GIN), National Guideline Clearinghouse (NGC), American Society of Clinical Oncology (ASCO), National Comprehensive Cancer Network (NCCN), National Institute for Health and Care Excellence (NICE), Registered Nurses’ Association of Ontario (RNAO), Australian Clinical Practice Guidelines (ACPG), Scottish Intercollegiate Guidelines Network (SIGN), Oncology Nursing Society (ONS), European Society for Medical Oncology (ESMO), Multinational Association of Supportive Care in Cancer (MASCC), American Society for Radiation Oncology (ASTRO), European Society for Radiotherapy and Oncology (ESTRO), Yimaitong, and the Chinese Medical Association. Additionally, comprehensive English and Chinese databases such as PubMed, Embase, The Cochrane Library, Web of Science, CINAHL, CNKI, VIP, Wanfang, and CBM were searched. To identify further eligible literature, we manually screened the reference lists of included studies and relevant reviews. The search was conducted using a combination of subject headings and free-text words.The English search terms used were: “Head and Neck Neoplasms/Head and Neck Cancer/Tongue Neoplasms/Laryngeal Neoplasms/Nasopharyngeal Carcinoma/Mouth Neoplasms” AND “Radiodermatitis/radiation-induced skin reaction (RISR)/radiation dermatitis/radiation injuries/radiation skin lesion” AND “Guideline/Evidence-Based Nursing/Evidence-Based Medicine/Best Practices/Recommended Practice/Evidence Summary/Systematic Review/Meta-Analysis.” Search strategies were adapted for each database’s specific requirements, covering the period from January 1, 2000, to August 31, 2024.We used the search strategy described in the **Supplementary Material 2**.

### 2.2 Inclusion and exclusion criteria for literature

#### 2.2.1 Inclusion criteria

Inclusion criteria for this study were:(1)study subjects were HNC patients aged ≥18 years undergoing radiotherapy;(2) their content explicitly addressed guidance on radiation dermatitis self-management, encompassing specific preventive and management measures;(3) Eligible document types included guidelines, clinical decision support tools, best practice manuals, evidence summaries, systematic reviews, and expert consensus statements;(4) Publications were limited to either Chinese or English.

#### 2.2.2 Exclusion criteria

Exclusion criteria were:(1) duplicated or translated publications; (2) literature for which the full text was unavailable; (3) outdated guideline documents that had been superseded by updated versions;(4) studies assessed as having low methodological quality.

### 2.3 Quality evaluation of the literature

Two researchers, both trained in evidence-based practice, independently completed the quality appraisal. Any discrepancies were resolved by a third-party expert in evidence-based nursing. Guidelines were appraised using the Appraisal of Guidelines for Research and Evaluation II (AGREE II) instrument (21). Their recommendations were categorized into three grades: Grade A if all six domains scored ≥ 60%; Grade B if ≥ 3 domains scored ≥ 30% while having at least one domain < 60%; and Grade C if ≥ 3 domains scored < 30%. Inter-rater agreement for the appraisals was assessed using the intraclass correlation coefficient (ICC), with an ICC > 0.75 indicating high consistency (22). Systematic reviews were appraised according to the A Measurement Tool to Assess Systematic Reviews-II (AMSTAR-II) (23). Expert consensus statements were evaluated using the Australian JBI Evidence-Based Health Care Center tool (24). Finally, clinical decision and evidence summaries were quality-appraised based on the original research’s study type, following the corresponding appraisal criteria from the JBI Manual for Evidence Synthesis (25).

### 2.4 Evidence extraction

Two researchers independently extracted data from the included literature, encompassing basic publication details (e.g., publication year, authors, article title and the evidence content). The extracted data were then cross-checked by both researchers; any disagreements were resolved by consensus or, if necessary, by adjudication from a third-party expert in evidence-based nursing. During the evidence synthesis process, conflicting evidence was handled by prioritizing the most recent and authoritative literature, followed by evidence-based sources, and then high-quality evidence.

### 2.5 Data synthesis

The collected data were classified and integrated by two researchers. When the content of the evidence is the same, give priority to the expression of professional and easy-toto-understand evidence; when the content of evidence complements each other, it can be integrated into an evidence information according to the logical relationship of evidence; when the conclusions of the evidence from various sources are controversial, the basic principles are that evidence-based evidence is preferred, high-quality evidence is preferred, the latest published authoritative evidence is preferred, and it is considered in combination with whether it is applicable to the self-management of radiation dermatitis in HNC patients.

### 2.6 Evidence level evaluation process

All evidence was used in the JBI Evidence-Based Health Care Center Evidence Grading and Recommended Levels of Evidence System (2014) (26), and the included evidence levels were divided into 1 to 5, with 1 being the highest level and 5 being the lowest level.

## 3 Result

### 3.1 Search result

**Figure 1:**
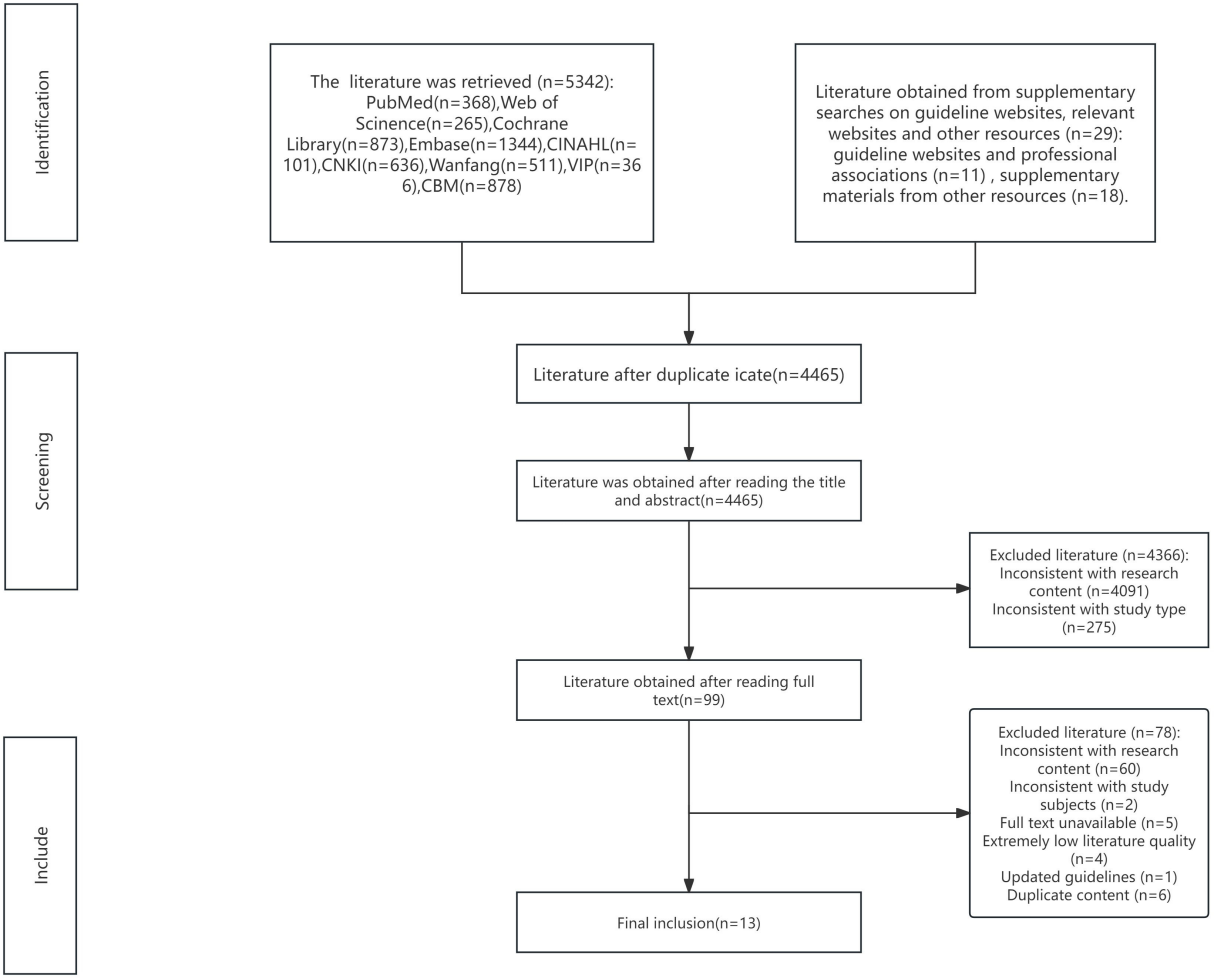
Flow chart of literature screening.

A total of 5,371 articles were initially retrieved through keyword searches and supplemental resources. After exclusion of 906 duplicate records, 4,465 articles were removed after title and abstract screening, and an additional 78 articles were excluded after full-text review. Ultimately, 21 articles were included: these comprised 7 guidelines (8, 27–32), 6 expert consensus statements (33–38), 2 evidence summaries (14, 39), 2 recommended practices (40, 41), 2 clinical decision support tools (42, 43), and 2 systematic reviews (44, 45). The literature screening process is illustrated in **Figure 1**, and the general characteristics of the included articles are presented in **Table 1**.

**Table 1:** Characteristics of the included articles (*N* = 21).

Included literature	Public-ation time (year)	Literature resource	Literature theme	Type of evidence
TARA et al. (27)	2023	Yimaitong	2023 MASCC Clinical Practice Guidelines: Prevention and Management of Acute Radiation Dermatitis	Guideline
Chinese Alliance for Radiation Oncology et al. (8)	2023	CNKI	Clinical Practice Guidelines for Prevention and Treatment of Radiation Dermatitis	Guideline
Agbejule et al. (28)	2021	ISNCC	Evidence-Based Guidelines for Prevention and Management of Radiation Dermatitis	Guideline
Tracy et al. (29)	2020	ONS	Guidelines for Cancer Treatment-Related Radiation Dermatitis	Guideline
Bennet et al. (30)	2020	SCoR	Radiation Therapy: Guidelines for Radiation Dermatitis for Healthcare Professionals	Guideline
Post et al. (31)	2018	BCCA	Symptom management guidelines:radiation dermatitis	Guideline
Harris et al. (32)	2015	NICE	Clinical Practice Guidelines:Skin care advice for patients undergoing radical external Beam megavoliage radiotherapy	Guideline
Shi chun meng et al. (33)	2024	CNKI	Expert Consensus on Diagnosis and Treatment of Radiation-Induced Skin Injury	Expert consensuses
Zhao chong et al. (34)	2018	Yimaitong	Expert Consensus on Nutrition and Supportive Therapy for Head and Neck Cancer Patients Undergoing Radiotherapy	Expert consensuses
Pinto et al. (35)	2016	PubMed	Management of Skin Reactions During Cetuximab Treatment in Association With Chemotherapy or Radiotherapy Update of the Italian Expert Recommendations	Expert consensuses
Zhu et al. (36)	2016	PubMed	Asian Experts’ Recommendations on Management of Skin and Mucosal Effects During Radiotherapy for Head and Neck Squamous Cell Carcinoma	Expert consensuses
Russi et al. (37)	2015	PubMed	Management of Acute Cutaneous Toxicity in Head and Neck Cancer Patients Treated with Radiotherapy, Chemotherapy, or EGFR Inhibitors	Expert consensuses
Blanchard et al. (38)	2014	PubMed	Management of somatic pain induced by head and neck cancer treatment:Pain following radiation therapy and chemotherapy.	Expert consensuses
Wang yuan yuan et al. (14)	2024	CNKI	Best Evidence Summary for Prevention and Management of Radiation Dermatitis in Patients with Head and Neck Tumors	Evidence summary
Wang qian (39)	2020	CNKI	Evidence Summary for Prevention and Management of Radiation Dermatitis	Evidence summary
JBI (40)	2021	JBI	Radiation Therapy (Skin Changes): Management	Recommended practice
JBI (41)	2021	JBI	Radiation Therapy (Skin Changes): Prevention	Recommended practice
Thomas et al. (42)	2024	Up To Date	Management and Prevention of Complications During Initial Treatment of Head and Neck Cancer	Clinical decision
Julie et al. (43)	2023	Up To Date	Radiation dermatitis	Clinical decision
Chan et al. (44)	2023	PubMed	Prevention of radiation dermatitis with skin hygiene and washing:a systematic review and meta-analysis	Systemic review
Kao et al. (45)	2022	PubMed	Topical Prevention of Radiation Dermatitis in Head and Neck Cancer Patients:A Network Meta-analysis	Systemic review

### 3.2 Quality evaluation results of the included literature

#### 3.2.1 Quality evaluation results of guidelines

This study included a total of seven guidelines. Their standardized domain scores and recommendations are presented in **Table 2**. The ICC for agreement were 0.807, 0.986, 0.904, 0.916, 0.606, 0.885, and 0.637, respectively, indicating good consistency for all and warranting their inclusion.

**Table 2:** Methodological evaluation of the guidelines included in this study (*N* = 7).

Included literature	Standardized Scores in Various Domains (%)						≥60%	≤30%	Quality Evaluation
	Scopes and objects	Stakeholder Particip-ant	Rigour of development	Clarity of guidelines	Applicability	Editorial independence			
TARA et al. (27)	91.67%	66.67%	70.31%	72.22%	33.33%	100%	5	1	B
Chinese Alliance for Radiation Oncology et al. (8)	83.33%	62.50%	12.50%	93.30%	26.04%	50.00%	3	2	B
Agbejule et al. (28)	88.89%	62.50%	68.23%	75.00%	20.83%	68.75%	5	1	B
Tracy et al. (29)	97.22%	84.72%	77.08%	83.33%	63.54%	89.58%	6	0	A
Bennet et al. (30)	95.83%	94.44%	86.98%	93.06%	88.54%	95.83%	6	0	A
Post et al. (31)	34.72%	33.33%	10.94%	61.11%	50.00%	10.41%	1	2	B
Harris et al. (32)	91.67%	88.89%	74.48%	62.50%	81.25%	95.83%	6	0	A

#### 3.2.3 Quality evaluation results of expert consensus

This study included six expert consensus statements. The studies by the Chinese Geriatric Society Burns Branch et al. (33) and Zhu et al. (36) were fully included as all appraisal items were rated “Yes,” indicating clear viewpoints. The studies by Zhao et al. (34), Russi et al. (37), and Blanchard et al. (38) were deemed of high overall quality and included, with only item 6, “Are the proposed opinions inconsistent with previous literature?”, rated as “Unclear.” Conversely, Pinto et al.’s study (35) was cautiously included, as item 1, “Is the source of the opinion clearly indicated?”, was rated “No,” and item 6, “Are the proposed opinions inconsistent with previous literature?”, was rated “Unclear.”

#### 3.2.4 Quality evaluation of clinical decisions, recommended practices and evidence summaries

This study included two recommended practices (40, 41), two clinical decisions (42, 43), and two evidence summaries (14, 39). The two clinical decisions originated from Up To Date, and the two recommended practices were sourced from JBI; both are reputable databases, allowing for direct inclusion of their extracted evidence. A total of six pieces of evidence were extracted from the two evidence summaries, all of which were included due to their high overall quality.

#### 3.2.5 Quality evaluation results of systematic reviews

This study included two systematic reviews. Kao et al.’s study (45) featured a complete design and received an overall AMSTAR-II quality rating of “high”, thus warranting its full inclusion. Conversely, Chan et al.’s study (44) had one critical item not met, accompanied by (or without) non-critical items rated as “unclear” or “no”, resulting in an overall AMSTAR-II quality rating of “low”. Nevertheless, given its generally reasonable study design, both researchers discussed and decided to cautiously include it.

#### 3.2.6 Summary of evidence

Following the extraction and synthesis of evidence from literature on self-management of radiation dermatitis in head and neck cancer patients, a total of 56 pieces of evidence were ultimately derived and categorized into six distinct themes.For detailed content, refer to **Table 3**.

**Table 3:** Evidence summary for self-management of radiodermatitis in head and neck cancer patients.

Evidence item		Evidence content	Evidence level
learning relevant knowledge	learning content	1.Learn and master knowledge related to radiation dermatitis, including its occurrence range, pathophysiology, incidence rate, grading, clinical manifestations, risk factors, and prevention strategies, etc. (30)	5
daily life management	quiting smoking	2.avoid smoking (30, 32, 37, 42).	5
	hygiene	3.It is recommended to clean the skin with warm water (at a temperature of 38-40°C) and mild, unscented, neutral or non-alkaline soap and/or unscented liquid body wash, no more than twice a day. For those with sensitive skin or wet desquamation, only clean with clear water. After cleaning, it is advisable to pat dry the irradiated skin with a soft, highly absorbent cotton towel (14, 29–32, 41, 44). 4.Keep the skin in the radiotherapy area exposed, clean and dry. Also, avoid sweating after radiotherapy to prevent skin irritation and reduce the risk of infection (8, 36, 43). 5.It is recommended that patients undergoing head radiotherapy use mild shampoo products for head cleaning. After shampooing, the hair should be air-dried naturally, and the use of a hair dryer for heat drying should be avoided (14, 30, 32). 6.keep nails trimmed regularly (35). 7.It is recommended that patients in need use antiperspirants or deodorants (unless they irritate the skin). When blisters or desquamation appear on the skin, their use should be discontinued (8, 29–32).	1 5 5 5 1
	moisturization	8.It is recommended to apply unscented, lanolin-free water-based moisturizers to intact skin 2–3 times a day, including on weekends when radiotherapy is not performed. When applying, the action should be gentle, and ensure that the moisturizer is completely absorbed (30, 41, 43). 9.If the moisturizer irritates the skin, or if blisters or desquamation appear on the skin, its use should be discontinued, and advice from professionals should be sought (30, 41, 43). 10.Avoid applying moisturizers or lotions 1 to 4 hours before radiotherapy to prevent the “accumulation” effect (8, 14, 30, 37).	5 5 5
	dressing	11.It is recommended to wear low-necked, loose-fitting, soft fabric or cotton clothes to avoid traumatic shearing and friction injuries. Low-necked tops and cotton T-shirts are recommended, while shirts, sweaters, etc., should be avoided (8, 31, 32, 38, 41, 43).	5
	avoiding sun exposure	12.During radiotherapy and the skin healing period, the irradiated skin should be protected from sunlight exposure. When going out, it is recommended to take physical sun protection measures, such as wearing a wide-brimmed protective hat during outdoor exposure (8, 30–32, 35, 37, 38, 41, 43). 13.It is recommended that patients undergoing neck radiotherapy use pure cotton or silk protective fabrics (such as silk scarves) to cover the neck treatment area when going out, so as to reduce ultraviolet radiation and external mechanical stimulation (30). 14.After radiotherapy and skin healing, the irradiated area should continue to be protected from sunlight for at least one year (30). 15.When exposure to sunlight is unavoidable, it is recommended to use sunscreen (with a sun protection factor of 30 or higher) on intact skin (30–32, 35).	1 5 5 5
	swimming	16.When there are no blisters or desquamation on the skin, swimming can be continued, but swimming in lakes, chlorinated pools, or hot tubs should be avoided (29, 30, 41). 17.Shower immediately after swimming and apply moisturizer (30, 31). 18.If swimming irritates the skin, or if blisters or desquamation appear on the skin in the radiotherapy area, swimming should be stopped (30, 31).	5 5 5
	shaving	19.It is recommended to minimize shaving during radiotherapy, and in particular, wet shaving with a razor on the treatment area should be avoided. If there are other needs, consult a professional medical staff (30, 32). 20.Avoid using straight-edge razors; it is recommended to use electric razors to remove local hair (31, 32, 43). 21.Avoid using pre-shave liquids, alcohol-containing aftershaves, waxes, or other hair removal creams (30–32, 35).	5 1 5
	avoiding friction	22.Avoid or reduce friction, scratching, and massaging of the skin in the radiotherapy area (8, 30–32, 36, 43). 23.Avoid wearing jewelry or using bandages, band-aids, tape, and adhesives on the radiotherapy area (30–32, 37, 41).	5 1
	avoiding irritation	24.Avoid using alcohol, cosmetic hair dyes, perfumes, and cosmetics or skincare products containing ethanol on or near the radiotherapy area (8, 30, 35, 38, 41, 43).	5
		25.Avoid using baby powder and cornstarch in skin folds (8, 41, 43). 26.Avoid applying heat or cold directly to the radiotherapy area, such as ice packs and heating pads (30–32). 27.Avoid damaging the skin in the irradiated area with sharp objects (30, 33).	5 5 5
self-monitoring	content of monitoring	28.For grade 1 radiodermatitis, one should self-monitor the head and neck dermatitis in terms of its location, size of the affected area, color, discomfort (such as burning sensation, itching, pulling sensation, tenderness), and erythema (31). 29.For grade 2 and above radiodermatitis, attention should also be paid to moist and dry areas, the size of the affected area, wound conditions, exudate (type, amount, odor), discomfort (burning sensation, itching, pulling sensation, tenderness), and clinical signs of infection (31). 30.Close observation should be paid to the neck shape, neck skin folds, skin moisture and sweating, etc. (8, 14, 30)	5 5 5
	frequency of monitoring	31.Continuous and regular monitoring of skin reactions should be conducted. During the initial stage of treatment, close monitoring should be performed once a week. As symptoms appear, the monitoring frequency should be gradually increased. For example, when erythema occurs, monitoring should be done at least twice a week (14, 36, 43).	5
	method of monitoring	32.It is recommended to document any differences in the skin through photographs, so that they can be promptly shared with radiation technologists and clinical medical staff (14, 30).	1
symptom management	infection management	33.The skin in the radiotherapy area should be kept intact. Hands should be washed and hand hygiene should be maintained before touching the radiotherapy area (14). 34.When blisters appear, self-treatment should be avoided to prevent infection (41). 35.In case of grade 1 acute radiation dermatitis, it is recommended to use corticosteroid cream as prescribed by the doctor to reduce inflammation, once or twice a day (31). 36.When a suspected wound infection occurs, it is recommended to clean the wound with normal saline as directed by a doctor (30, 41). 37.When a wound infection occurs, systemic and/or topical antibacterial drugs should be used as prescribed by a doctor (8, 30, 32, 33, 43). 38.It is recommended to use appropriate dressings on broken skin to reduce further trauma and infection. If there is evidence of infection, silver sulfadiazine dressings can be used topically as prescribed by a doctor (28, 31–33). 39.For severe desquamation, it is recommended to inspect and change dressings daily, and observe the wound (with gentle wiping if necessary) to prevent infection, especially in skin folds (40).	5 5 1 5 4 2 5
	pain management	40.Follow the doctor’s advice to use analgesics to reduce the pain level of patients with radiation-induced skin damage (30, 33, 40). 41.Pain can be auxiliarily relieved through methods such as relaxation or hypnosis (38).	4 5
	pruritus management	42.Avoid scratching the skin in the radiotherapy area (41). 43.It is recommended to pat the local skin gently to relieve itching (30). 44.For severe itching, it is recommended to use a low-dose corticosteroid cream on intact skin 1–2 times a day as prescribed by a doctor. At the same time, care should be taken to avoid excessive use to prevent the skin from thinning. If an allergic reaction occurs, the use should be discontinued immediately (27, 31, 33, 40).	5 5 5
	desquamation management	45.When grade 1 radiodermatitis only presents as dry desquamation without other accompanying symptoms, no treatment may be necessary (42, 43). 46.For radiodermatitis of grade 2 or higher, which may present with moist desquamation, it is recommended to dress the wound locally with semipermeable, low-adherent, or non-adherent dressings, such as hydrogel or hydrocolloid dressings (29, 31, 40).	1 1
dietary management	dietary principles	47.It is recommended to drink 6–8 cups of water daily to keep the skin hydrated (30, 31). 48.For patients with neck tumors undergoing radiotherapy, the recommended daily calorie intake is 25–30 kcal/kg. If the patient has severe complications, the recommended daily calorie intake is 30–35 kcal/kg (34). 49.HNC patients undergoing radiotherapy should appropriately increase the proportion of fat in energy supply; the protein supply is 1.0–1.5 g/kg (34). 50.During the wound healing phase, one should consume foods rich in various nutrients and vitamins, such as fruits, vegetables, whole grains, and lean protein (30, 31).	5 2 2 5
	weight management	51.Ensure adequate nutrient intake and avoid excessive weight loss. During radiotherapy, if there is a weight loss of more than 10% within 6 months, more than 5% within 3 months, or a continuous weight loss of 0.5 kg per week, inform medical staff in a timely manner (31, 34). 52.After radiotherapy, regular weight monitoring and follow-up are still necessary. For patients with malnutrition, they can consult nutrition experts (34, 37).	5 5
psychosocial support	management of emotions	53.Accept emotional changes. Anxiety and depression are common mental complications in HNC patients (33). 54.During radiotherapy, if you encounter difficulties, you should actively seek support and help from medical staff and family members to reduce confusion and anxiety (30).	5 5
	multidisciplinary team collaboration	55.Receive comprehensive treatment provided by a multidisciplinary team, including trauma surgeons, radiation oncologists, medical oncologists, dermatologists, nutritionists, and nursing staff, among others (42, 43).	5
	regular follow-up visits	56.Follow the doctor’s advice for regular follow-up visits. It is recommended that patients at risk of chronic radiodermatitis regularly return to the community or outpatient clinic for reexaminations (30, 43).	5

## 4 Discussion

### 4.1 Evidence synthesis process

This study rigorously adhered to recommended evidence-based nursing methodologies, systematically constructing a comprehensive evidence summary for radiation dermatitis. Following the “6S” evidence model, searches primarily focused on high-quality literature such as guidelines, clinical decisions, and expert consensus statements, mainly sourced from authoritative databases and websites including UpToDate, JBI, MASCC, and SCoR. After rigorous quality appraisal, 56 pieces of evidence were ultimately identified. The entire process was independently conducted by two postgraduate researchers, with head and neck radiotherapy clinical experts and evidence-based practice experts overseeing quality control and ensuring the accuracy of evidence content, thereby guaranteeing the scientific rigor of the evidence summary process. These pieces of evidence encompassed various aspects of HNC radiation dermatitis, including learning related knowledge, daily life management, self-monitoring, symptom management, dietary management, and psychosocial support. The research team critically appraised the evidence, thoroughly considering the clinical context when assessing its strengths and limitations. Compared to existing literature on radiation dermatitis prevention and management strategies (14, 39), the 56 pieces of evidence summarized in this study place a greater emphasis on a patient-centered philosophy, providing HNC patients with precise, scientific, and rational management guidance to enhance their self-management efficacy. Furthermore, these six themes offer a structured and comprehensive overview of current evidence and practical recommendations, contributing to a clearer understanding of the current state of self-management for radiation dermatitis in HNC patients.

### 4.2 Learning knowledge related to radiodermatitis is the foundation for self-management in HNC patients

Evidence statement 1 emphasizes the crucial role of acquiring comprehensive knowledge about radiation dermatitis (28). Research indicates (46–48) that providing HNC patients with comprehensive knowledge on radiation dermatitis fosters their health beliefs and promotes self-care behaviors, which are pivotal for effective self-management. Guidelines published by SCoR (30) repeatedly highlight the importance of patients understanding factors influencing the development and severity of radiation dermatitis, offering self-care advice to ensure patients have a reliable reference. Therefore, healthcare professionals should comprehensively assess patients’ cognitive abilities and understanding of their disease treatment. This assessment should be integrated with their specific clinical condition to provide individualized health education (32). Key areas of focus should include the scope, incidence, grading, clinical manifestations, and risk factors of radiation dermatitis to enhance patient awareness and strengthen their capacity and willingness for self-management. Furthermore, given the insidious onset and progressive nature of chronic radiation dermatitis (14), patients require long-term self-management well after discharge. Hence, healthcare professionals can disseminate knowledge about radiation dermatitis to patients via platforms such as WeChat groups, official accounts, mini-programs, and mobile apps, thereby facilitating better patient adherence to nursing care and promoting standardized management of radiation dermatitis (49).

### 4.3 Daily life management is an important part of self-management for HNC patients

Evidence statements 2 to 27 pertain to the daily management of radiation dermatitis, encompassing eight key areas: quiting smoking, hygiene, moisturizing, dressing, avoiding sun exposure, swimming, shaving, avoiding friction, and avoiding irritation.

Firstly, research indicates that continued smoking by HNC patients during or early after curative treatment exacerbates and prolongs mucosal reactions, while also impairing oncological outcomes.Therefore, before initiating radiotherapy, patients should be educated on the critical importance of smoking cessation and guided on effective quitting strategies, alongside avoiding secondhand smoke exposure to minimize further bodily harm (42, 50).

Secondly, studies confirm that proper skin management improves comfort during radiotherapy, reduces inflammation, and promotes healing of radiation dermatitis-affected skin areas (14, 51). Healthcare professionals should provide personalized guidance and recommendations to patients, considering their specific conditions, across various aspects including hygiene and moisturizing. Concurrently, patients and their families must fully understand essential skin care protocols, selecting appropriate moisturizing products and applying them correctly, and performing daily skin care under professional guidance (30).

Thirdly, radiotherapy increases skin sensitivity, making it vulnerable to ultraviolet(UV) radiation, which elevates the risk of skin damage and carcinogenesis. Thus, during radiotherapy and skin healing, effective sun protection measures are crucial for protecting the skin, reducing injury severity, and promoting healing (8, 31). Healthcare professionals should advise patients to strictly adhere to physical and chemical sun protection recommendations, select appropriate products, and employ proper protective measures. If skin issues arise, patients must promptly consult professionals for timely and appropriate management and advice (30–32, 35).

Finally, HNC patients’ neck skin and tissues become fragile and susceptible to friction-induced injury post-radiotherapy. Therefore, during treatment, patients should avoid unnecessary friction and irritation in the irradiated area to mitigate the risk of skin damage and infection (30, 31, 37, 41). Healthcare professionals must provide detailed guidance and education to help patients understand the importance of avoiding these actions and offer alternative methods to alleviate skin discomfort, thereby minimizing further harm to the treated skin. It is noteworthy that while some guidelines and clinical decisions mention evidence related to clothing, swimming, and shaving, health education in these areas currently relies heavily on clinical experience, with limited evidence on long-term patient outcomes. Future research is warranted to enhance the quality and reliability of evidence in these domains.

### 4.4 Self-monitoring is an important measure for the early prevention of radiodermatitis

Evidence statements 28 to 32 highlight the critical importance of patient self-monitoring for radiation dermatitis. Two specific references (30, 36) detail relevant aspects of this self-monitoring, including observing skin changes in the irradiated area, pain, pruritus, and signs of infection. Research indicates that approximately 87% of HNC patients undergoing radiotherapy develop radiation dermatitis, with acute forms typically emerging within 90 days of the first radiotherapy session or radiation exposure, and skin changes potentially appearing within hours (8, 37). Furthermore, radiation dermatitis associated with radiotherapy combined with cetuximab appears earlier and is more severe compared to radiotherapy alone or chemoradiotherapy, commonly presenting as marked dryness, severe inflammation, and epidermal necrosis (14, 38, 43). Consequently, patients and caregivers should be instructed to continuously monitor the irradiated skin for changes, pain, pruritus, and signs of infection from the onset of radiotherapy. This monitoring should be integrated with attention to BMI and blood glucose control (especially for diabetic patients) (40, 41), seeking specialized nutritional or diabetes support when necessary, to facilitate early detection and intervention.

### 4.5 Symptom management is key to improving the quality of life for HNC patients

Given the significant impact of radiation dermatitis on the quality of life for HNC patients, targeted and precise management of its symptoms is essential. Evidence statements 33 to 46 summarize relevant findings on radiation dermatitis symptom management, covering four key areas: infection, pain, pruritus, and desquamation control.

Firstly, infection is a common complication of radiation dermatitis, particularly prone to occurring when the skin is broken or ulcerated (52). Infections not only exacerbate patient suffering but can also lead to treatment interruptions, compromising treatment efficacy (30). Consequently, infection management is a vital component of radiation dermatitis symptom control. Key measures to safeguard patient skin health include maintaining skin integrity in the irradiated area, practicing proper hand hygiene, correctly managing blisters, promptly treating wound infections, and providing appropriate care for severe moist desquamation (14, 30, 41). Healthcare professionals should offer tailored nursing advice and guidance based on the patient’s specific condition, such as instructing them to closely monitor skin changes, keep the skin clean and dry, and use antibiotics as prescribed.

Secondly, pain caused by radiation dermatitis significantly diminishes patients’ quality of life, affecting their daily activities and sleep (30). The objective of pain management is to alleviate discomfort and enhance patient comfort (53). Pain control for patients with radiation-induced skin injury necessitates a comprehensive approach incorporating both pharmacological and non-pharmacological methods. For instance, analgesic medications, when used as prescribed, can effectively reduce pain, though attention to side effects and individualized adjustments is crucial (30, 33, 40). Non-pharmacological methods like relaxation or hypnosis can serve as complementary strategies, aiding patients in coping with pain and improving their quality of life (38). Healthcare professionals should formulate personalized pain management plans based on individual patient circumstances, providing corresponding guidance and support.

Thirdly, pruritus is a common symptom of radiation dermatitis, particularly pronounced during the acute phase (8). It not only impacts patients’ mood and sleep but can also lead to scratching, which may cause skin breakage (30). For mild cases, patients can be advised to gently pat the affected area to relieve itching (30, 41). For severe pruritus, low-dose corticosteroid creams may be used under a physician’s guidance, though careful attention to application methods, dosage, and avoidance of overuse or allergic reactions is necessary (29, 40). Healthcare professionals should provide detailed guidance and education to help patients better manage skin pruritus issues during radiotherapy.

Finally, desquamation resulting from radiation dermatitis not only affects patients’ appearance but can also lead to skin dryness, itching, and pain (54). The goal of desquamation management is to alleviate skin dryness, promote skin repair, and reduce discomfort associated with flaking (55). Management approaches for radiation dermatitis should vary according to its severity. Grade 1 radiation dermatitis primarily focuses on moisturizing and avoidance strategies (42). For Grade 2 or higher radiation dermatitis, appropriate dressings are required to protect the wound bed, promote healing, and prevent infection (31, 40). Throughout the entire care process, patients should be strictly guided to follow their physician’s recommendations and undergo regular wound assessment and care.

### 4.6 Optimizing diet can effectively reduce the risk of developing radiodermatitis

Evidence statements 47 to 52 highlight the crucial role of a healthy, balanced diet in preventing and managing radiation dermatitis. Extensive research indicates that obesity, malnutrition, and diabetes are significant risk factors for radiation dermatitis (30, 32). Obese patients, with greater adipose tissue, may experience heightened inflammatory responses during radiotherapy, exacerbating skin damage (32). Malnutrition, conversely, compromises skin barrier function, impairing its intrinsic self-repair capacity (56). Diabetic patients, due to suboptimal glycemic control, are more prone to skin infections and delayed healing (30). Therefore, radiotherapy and nutrition specialists should provide patients with scientific dietary advice, detailing daily requirements for energy, fat, protein, and water, advising on suitable food choices and dietary patterns, and guiding rational eating to enhance skin resilience (34). Furthermore, diabetic patients must be educated on proper blood glucose monitoring and control (30). However, the current evidence primarily stems from expert opinions and practical experience (34), resulting in a lower level of evidence quality. Future high-quality studies, such as randomized controlled trials and prospective cohort studies, are necessary to further validate the efficacy of dietary interventions in preventing and treating radiation dermatitis, thereby providing HNC patients with more robust and reliable nutritional support strategies.

### 4.7 Good psychosocial support can promote the rehabilitation of HNC patients

Evidence statements 53 to 56 underscore the importance of psychosocial support. Research indicates that HNC patients report a high incidence of psychosocial distress during radiotherapy, with severity increasing as treatment progresses. This distress impairs their capacity to effectively cope with cancer, physical symptoms, and treatment, profoundly impacting their quality of life (57). Addressing psychosocial distress in HNC is crucial for improving the quality of life for both patients and their caregivers; experts recommend integrating psychosocial interventions into HNC patient care pathways at an early stage (58). Consequently, during radiotherapy, healthcare professionals should provide HNC patients with comprehensive support and care, encompassing psychological support, multidisciplinary integrated treatment, and regular follow-up visits. Acknowledging emotional changes and actively seeking support can help patients better cope with psychological stress (42). Furthermore, multidisciplinary team collaboration provides comprehensive healthcare services and enhances treatment efficacy (30, 33, 43), while regular follow-up facilitates timely problem detection and management, ensuring the patient’s recovery process (30). Patients should adhere actively to healthcare professionals’ advice, confronting the disease alongside their families to collectively facilitate recovery.

### 4.8 Limitations

Firstly, the evidence synthesis process in this study was limited to Chinese and English literature, excluding publications in other languages. Additionally, current research on radiation dermatitis self-management remains limited, with most studies lacking in-depth investigation into specific self-management components, resulting in a narrow scope and insufficient targeting. Moreover, existing guidelines and expert consensuses often lack specific guidance on psychological support for HNC patients. Future research should expand into the psychosocial support domain to alleviate the adverse effects of negative emotions. Given the variability in healthcare settings across different regions, some evidence may be challenging to implement in practice. Therefore, when applying this evidence, consideration must be given to the national context, patients’ cultural backgrounds, health literacy, and lifestyle habits, alongside local healthcare resources and economic capacity, to develop contextually appropriate self-management intervention protocols for radiation dermatitis in local HNC patients.

## 5 Conclusion

This study synthesized 56 evidence items across six themes related to self-management of radiation dermatitis in HNC patients. Specifically, these themes include learning relevant knowledge, daily life management, self-monitoring, symptom management, dietary management, and psychosocial support. Collectively, this evidence provides a crucial foundation for healthcare professionals in educating and guiding patients in self-management, thereby facilitating the standardization of patients’ self-management practices. Furthermore, the effective delivery of this guidance is best achieved through a collaborative, multidisciplinary team approach to ensure comprehensive patient care. Looking forward, future high-quality studies, such as randomized controlled trials, are essential to further validate and strengthen the evidence for these self-management interventions.

## Data Availability

The original contributions presented in the study are included in the article/**Supplementary Material**. Further inquiries can be directed to the corresponding author.
