# Supplementary material for: Best evidence summary for self-management of radiodermatitis in head and neck cancer patients: an integrative review
Source: Front Oncol. 2025 Nov 13;15:1693170. doi: 10.3389/fonc.2025.1693170 (PMC12658905; doi:10.3389/fonc.2025.1693170)
Supplement: Supplementary file 1 [file DataSheet1.docx]

**1 The Production Process of Evidence Summary**

There is currently a lack of standardized processes for the production of evidence summaries. Fudan University Center for Evidence-based Nursing draws on the methodology of the Joanna Briggs Institute (JBI) Evidence Based Healthcare Center in Australia to produce evidence summaries, and proposes a suggested process for producing evidence summaries during the evidence conversion process, as shown in Figure 1.


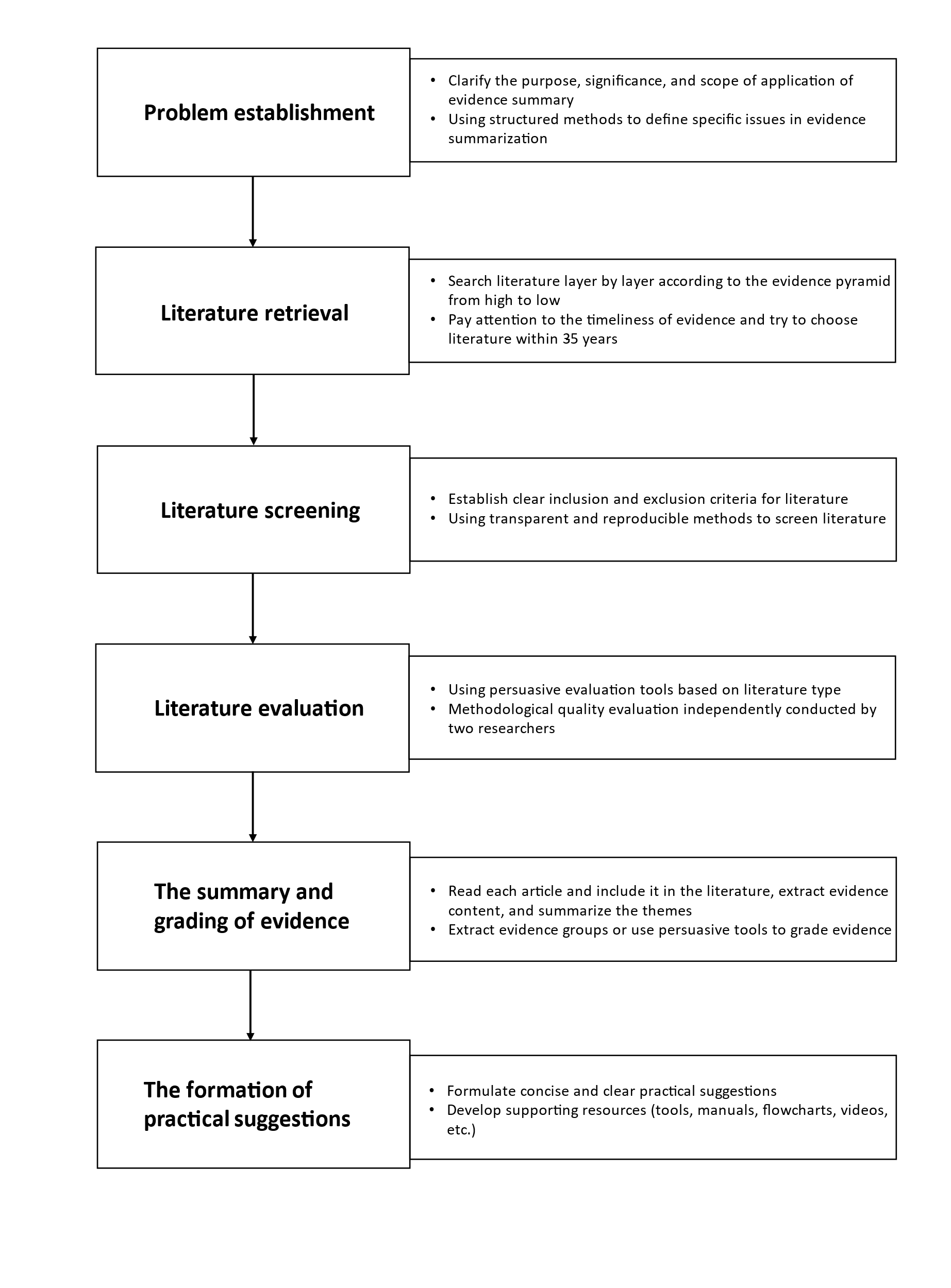


FIGURE 1 The Production Process of Evidence Summary

**2 Suggestions for the Report Summarizing Evidence**

At present, there is a lack of reporting standards for summarizing evidence. The Fudan University Center for Evidence-based Nursing has analyzed the summary of published evidence summaries both domestically and internationally, suggesting that the report on evidence summaries should be structured into the following five parts.

**2.1 Background:**

Report the clinical background of the evidence summary, explain the purpose and significance of the evidence summary, and declare the scope of application.

**2.2 Methods:**

Report on the structured PICOs framework of the evidence summary, describe the search database and search terms, outline the inclusion and exclusion criteria for the reported evidence, explain the literature quality evaluation methods and tools, elaborate on the evidence aggregation and grading methods, and describe the qualifications of the production team, including any potential conflicts of interest.

**2.3 Results:**

Report the basic information of the evidence, including the results of evidence retrieval and screening, as well as the sources, types, and themes of the evidence; Report on the methodological quality evaluation results of the incorporated evidence; Present each piece of evidence and its level either in text or in tabular form, indicating the source. Offer evidence-based practical recommendations in a concise and clear manner.

**2.4 Discussion：**

Discuss the significance and implications of summarizing evidence for practice, analyze the methodological limitations that affect the authenticity and generalizability of the evidence, and provide suggestions or countermeasures.

**2.5 Others：**

Include appendices as needed, such as methodological support materials like evidence retrieval and screening flowcharts, basic feature tables for included evidence, summary tables of evidence content, sources, and levels, as well as supporting practical resources like evaluation tools, practice manuals, flowcharts, and video resources.
