# Supplementary material for: Best evidence summary for self-management of radiodermatitis in head and neck cancer patients: an integrative review
Source: Front Oncol. 2025 Nov 13;15:1693170. doi: 10.3389/fonc.2025.1693170 (PMC12658905; doi:10.3389/fonc.2025.1693170)
Supplement: Supplementary file 2 [file DataSheet2.docx]

****Search Strategy****

**1 CNKI 636**

#1 TKA = Head and neck tumors + Upper aerodigestive tract tumors + Head tumors + Neck tumors + Head cancer + Neck cancer + Cervical cancer + Head and neck cancer + Upper aerodigestive tract (UADT) tumors + Head and neck malignant tumors + Head and neck malignancy + Nasopharyngeal tumors + Nasopharyngeal cancer + Nasal cancer + Laryngeal tumors + Larynx tumors + Laryngeal cancer + Hypopharyngeal cancer + Oral tumors + Mouth tumors + Oral cavity cancer + Mouth cancer + Oral cancer + Hypopharyngeal tumors + Oropharyngeal tumors + Oropharyngeal cancer

#2 TKA = Radiation dermatitis + Radiation-induced skin injury + Radiation-induced skin reaction + Radiation-induced dermatitis + Radiation recall dermatitis + Radiation recall reaction#3 #1 AND #2

**2 Wanfang 511**

#1 Title or Keywords: (Head and neck tumors or Upper aerodigestive tract tumors or Head tumors or Neck tumors or Head cancer or Neck cancer or Cervical cancer or Head and neck cancer or UADT tumors or Head and neck tumors or Head and neck malignant tumors or Head and neck malignancy or Nasopharyngeal tumors or Nasopharyngeal cancer or Nasal cancer or Laryngeal tumors or Larynx tumors or Laryngeal cancer or Hypopharyngeal cancer or Oral tumors or Mouth tumors or Oral cavity cancer or Mouth cancer or Oral cancer or Hypopharyngeal tumors or Oropharyngeal tumors or Oropharyngeal cancer)

#2 Title or Keywords: (Radiation dermatitis or Radiation-induced skin injury or Radiation-induced skin reaction or Radiation-induced dermatitis or Radiation recall dermatitis or Radiation recall reaction)

#3 #1 AND #2

**3 VIP 366**

#1 Title or Keywords: (Head and neck tumors or Upper aerodigestive tract tumors or Head tumors or Neck tumors or Head cancer or Neck cancer or Cervical cancer or Head and neck cancer or UADT tumors or Head and neck tumors or Head and neck malignant tumors or Head and neck malignancy or Nasopharyngeal tumors or Nasopharyngeal cancer or Nasal cancer or Laryngeal tumors or Larynx tumors or Laryngeal cancer or Hypopharyngeal cancer or Oral tumors or Mouth tumors or Oral cavity cancer or Mouth cancer or Oral cancer or Hypopharyngeal tumors or Oropharyngeal tumors or Oropharyngeal cancer)

#2 Title or Keywords: (Radiation dermatitis or Radiation-induced skin injury or Radiation-induced skin reaction or Radiation-induced dermatitis or Radiation recall dermatitis or Radiation recall reaction)

#3 #1 AND #2

**4 CBM 878**

#1 "Head and neck tumors"[Unweighted: Expanded]
#2 "Upper aerodigestive tract tumors"[Common Fields: Smart] OR "Head tumors"[Common Fields: Smart] OR "Neck tumors"[Common Fields: Smart] OR "Head cancer"[Common Fields: Smart] OR "Neck cancer"[Common Fields: Smart] OR "Cervical cancer"[Common Fields: Smart] OR "Head and neck cancer"[Common Fields: Smart] OR "UADT tumors"[Common Fields: Smart] OR "Head and neck tumors"[Common Fields: Smart] OR "Head and neck malignant tumors"[Common Fields: Smart] OR "Head and neck malignancy"[Common Fields: Smart] OR "Nasopharyngeal cancer"[Common Fields: Smart] OR "Nasal cancer"[Common Fields: Smart] OR "Laryngeal tumors"[Common Fields: Smart] OR "Laryngeal cancer"[Common Fields: Smart] OR "Hypopharyngeal cancer"[Common Fields: Smart] OR "Mouth tumors"[Common Fields: Smart] OR "Oral cavity cancer"[Common Fields: Smart] OR "Mouth cancer"[Common Fields: Smart] OR "Oral cancer"[Common Fields: Smart] OR "Hypopharyngeal cancer"[Common Fields: Smart] OR "Oropharyngeal cancer"[Common Fields: Smart]

#3 #1 OR #2

#4 "Radiation dermatitis"[Unweighted: Expanded]
#5 "Radiation-induced skin reaction"[Common Fields: Smart] OR "Radiation-induced skin injury"[Common Fields: Smart] OR "Radiation-induced dermatitis"[Common Fields: Smart] OR "Radiation recall dermatitis"[Common Fields: Smart] OR "Radiation recall reaction"[Common Fields: Smart]

#6 #4 OR #5

#7 #3 AND #6

**5 PubMed 368**

#1 "Head and Neck Neoplasms"[MeSH Terms] OR "Nasopharyngeal Carcinoma"[MeSH Terms] OR "Laryngeal Neoplasms"[MeSH Terms] OR "Mouth Neoplasms"[MeSH Terms] OR "Oropharyngeal Neoplasms"[MeSH Terms] OR "Otorhinolaryngologic Neoplasms"[MeSH Terms]

#2 "Neoplasms, Head and Neck"[Title/Abstract] OR "Head, Neck Neoplasms"[Title/Abstract] OR "Head and Neck Neoplasm"[Title/Abstract] OR "Cancer of Head and Neck"[Title/Abstract] OR "Head and Neck Cancer"[Title/Abstract] OR "Cancer of the Head and Neck"[Title/Abstract] OR "Upper Aerodigestive Tract Neoplasms"[Title/Abstract] OR "Neoplasms, UADT"[Title/Abstract] OR "UADT Neoplasms"[Title/Abstract] OR "Upper Aerodigestive Tract Neoplasm"[Title/Abstract] OR "Head Neoplasms"[Title/Abstract] OR "Neoplasms, Head"[Title/Abstract] OR "Head Neoplasm"[Title/Abstract] "Neoplasm, Head"[Title/Abstract] OR "Neck Neoplasms"[Title/Abstract] OR "Neoplasms, Neck"[Title/Abstract] OR "Neck Neoplasm"[Title/Abstract] OR "Neoplasm, Neck"[Title/Abstract] OR "Cancer of Head"[Title/Abstract] OR "Head Cancers"[Title/Abstract] OR "Head Cancer"[Title/Abstract] OR "Cancer, Head"[Title/Abstract] OR "Cancers, Head"[Title/Abstract] OR "Cancer of the Head"[Title/Abstract] OR "Cancer of Neck"[Title/Abstract] OR "Neck Cancers"[Title/Abstract] OR "Neck Cancer"[Title/Abstract] OR "Cancer, Neck"[Title/Abstract] OR "Cancers, Neck"[Title/Abstract] OR "Cancer of the Neck"[Title/Abstract] OR "Carcinoma, Nasopharyngeal"[Title/Abstract] OR "Carcinomas, Nasopharyngeal"[Title/Abstract] OR "Nasopharyngeal Carcinomas"[Title/Abstract] OR "Neoplasms, Laryngeal"[Title/Abstract] OR "Laryngeal Neoplasm"[Title/Abstract] OR "Neoplasm, Laryngeal"[Title/Abstract] OR "Larynx Neoplasms"[Title/Abstract] OR "Larynx Neoplasm"[Title/Abstract] OR "Neoplasms, Larynx"[Title/Abstract] OR "Cancer of Larynx"[Title/Abstract] OR "Larynx Cancers"[Title/Abstract] OR "Laryngeal Cancer"[Title/Abstract] OR "Cancer, Laryngeal"[Title/Abstract] OR "Cancers, Laryngeal"[Title/Abstract] OR "Laryngeal Cancers"[Title/Abstract] OR "Larynx Cancer"[Title/Abstract] OR "Cancer, Larynx"[Title/Abstract] OR "Cancers, Larynx"[Title/Abstract] OR "Cancer of the Larynx"[Title/Abstract] OR "Mouth Neoplasm"[Title/Abstract] OR "Neoplasm, Mouth"[Title/Abstract] OR "Neoplasms, Oral"[Title/Abstract] OR "Neoplasm, Oral"[Title/Abstract] OR "Oral Neoplasm"[Title/Abstract] OR "Oral Neoplasms"[Title/Abstract] OR "Neoplasms, Mouth"[Title/Abstract] OR "Cancer of Mouth"[Title/Abstract] OR "Mouth Cancers"[Title/Abstract] OR "Oral Cancer"[Title/Abstract] OR "Cancer, Oral"[Title/Abstract] OR "Cancers, Oral"[Title/Abstract] OR "Oral Cancers"[Title/Abstract] OR "Cancer of the Mouth"[Title/Abstract] OR "Mouth Cancer"[Title/Abstract] OR "Cancer, Mouth"[Title/Abstract] OR "Cancers, Mouth"[Title/Abstract] OR "Oropharyngeal Neoplasm"[Title/Abstract] OR "Oropharynx Neoplasms"[Title/Abstract] OR "Oropharynx Neoplasm"[Title/Abstract] OR "Neoplasms, Oropharyngeal"[Title/Abstract] OR "Oropharnyx Cancer"[Title/Abstract] OR "Oropharyngeal Cancer"[Title/Abstract] OR "Cancer, Oropharyngeal"[Title/Abstract] OR "Cancers, Oropharyngeal"[Title/Abstract] OR "Oropharyngeal Cancers"[Title/Abstract] OR "Oropharynx Cancer"[Title/Abstract] OR "Cancer, Oropharynx"[Title/Abstract] OR "Cancers, Oropharynx"[Title/Abstract] OR "Oropharynx Cancers"[Title/Abstract] OR "Cancer of the Oropharynx"[Title/Abstract] OR "Otorhinolaryngologic Neoplasm"[Title/Abstract] OR "Otorhinolaryngological Neoplasms"[Title/Abstract] OR "Otorhinolaryngeal Cancer"[Title/Abstract] OR "Ear nose throat cancer"[Title/Abstract] OR "ENT cancer"[Title/Abstract] OR "ORL cancer"[Title/Abstract] OR "Cervicofacial cancer"[Title/Abstract]

#3 #1 OR #2

#4 "Radiodermatitis"[MeSH Terms]

#5 "Radiodermatitides"[Title/Abstract] OR "radiation induced dermatitis"[Title/Abstract] OR "radiation induced dermatitis"[Title/Abstract] OR "dermatitis radiation induced"[Title/Abstract] OR (("dermatiti"[All Fields] OR "Dermatitis"[MeSH Terms] OR "Dermatitis"[All Fields] OR "Dermatitides"[All Fields]) AND "Radiation-Induced"[Title/Abstract]) OR "dermatitis radiation induced"[Title/Abstract] OR ("Radiation-Induced"[All Fields] AND "Dermatitides"[Title/Abstract]) OR "radiation recall dermatitis"[Title/Abstract] OR (("dermatiti"[All Fields] OR "Dermatitis"[MeSH Terms] OR "Dermatitis"[All Fields] OR "Dermatitides"[All Fields]) AND "radiation recall"[Title/Abstract]) OR "dermatitis radiation recall"[Title/Abstract] OR ((("radiate"[All Fields] OR "radiated"[All Fields] OR "radiates"[All Fields] OR "radiating"[All Fields] OR "Radiation"[MeSH Terms] OR "Radiation"[All Fields] OR "electromagnetic radiation"[MeSH Terms] OR ("electromagnetic"[All Fields] AND "Radiation"[All Fields]) OR "electromagnetic radiation"[All Fields] OR "radiations"[All Fields] OR "radiation s"[All Fields] OR "radiator"[All Fields] OR "radiators"[All Fields]) AND ("mental recall"[MeSH Terms] OR ("mental"[All Fields] AND "Recall"[All Fields]) OR "mental recall"[All Fields] OR "Recall"[All Fields] OR "recalling"[All Fields] OR "recallable"[All Fields] OR "recalled"[All Fields] OR "recallers"[All Fields] OR "recalls"[All Fields])) AND "Dermatitides"[Title/Abstract]) OR "radiation recall reaction"[Title/Abstract] OR "radiation recall reactions"[Title/Abstract] OR "reaction radiation recall"[Title/Abstract] OR (("Reaction"[All Fields] OR "reaction s"[All Fields] OR "Reactions"[All Fields]) AND "radiation recall"[Title/Abstract]) OR "recall reaction radiation"[Title/Abstract] OR (("mental recall"[MeSH Terms] OR ("mental"[All Fields] AND "Recall"[All Fields]) OR "mental recall"[All Fields] OR "Recall"[All Fields] OR "recalling"[All Fields] OR "recallable"[All Fields] OR "recalled"[All Fields] OR "recallers"[All Fields] OR "recalls"[All Fields]) AND "reactions radiation"[Title/Abstract]) OR ("radiation dermatitis"[Title/Abstract] OR "radiation induced skin reaction*"[Title/Abstract] OR "RISR"[Title/Abstract] OR "ARD"[Title/Abstract] OR "radiation induced skin injur*"[Title/Abstract] OR "radiation induced skin toxicit*"[Title/Abstract] OR "radiation induced skin damage"[Title/Abstract])

#6 #4 OR #5

#7 #3 AND #6

**6 Web of Science 265**

#1 TS="Head and Neck Neoplasms" OR "Nasopharyngeal Carcinoma" OR "Laryngeal Neoplasms" OR "Mouth Neoplasms" OR "Oropharyngeal Neoplasms" OR "Otorhinolaryngologic Neoplasms" OR "Neoplasms, Head and Neck" OR "Head, Neck Neoplasms" OR "Head and Neck Neoplasm" OR "Cancer of Head and Neck" OR "Head and Neck Cancer" OR "Cancer of the Head and Neck" OR "Upper Aerodigestive Tract Neoplasms" OR "Neoplasms, UADT" OR "UADT Neoplasms" OR "Upper Aerodigestive Tract Neoplasm" OR "Head Neoplasms" OR "Neoplasms, Head" OR "Head Neoplasm" OR "Neoplasm, Head" OR "Neck Neoplasms" OR "Neoplasms, Neck" OR "Neck Neoplasm" OR "Neoplasm, Neck" OR "Cancer of Head" OR "Head Cancers" OR "Head Cancer" OR "Cancer, Head" OR "Cancers, Head" OR "Cancer of the Head" OR "Cancer of Neck" OR "Neck Cancers" OR "Neck Cancer" OR "Cancer, Neck" OR "Cancers, Neck" OR "Cancer of the Neck" OR "Carcinoma, Nasopharyngeal" OR "Carcinomas, Nasopharyngeal" OR "Nasopharyngeal Carcinomas" OR "Neoplasms, Laryngeal" OR "Laryngeal Neoplasm" OR "Neoplasm, Laryngeal" OR "Larynx Neoplasms" OR "Larynx Neoplasm" OR "Neoplasms, Larynx" OR "Cancer of Larynx" OR "Larynx Cancers" OR "Laryngeal Cancer" OR "Cancer, Laryngeal" OR "Cancers, Laryngeal" OR "Laryngeal Cancers" OR "Larynx Cancer" OR "Cancer, Larynx" OR "Cancers, Larynx" OR "Cancer of the Larynx" OR "Mouth Neoplasm" OR "Neoplasm, Mouth" OR "Neoplasms, Oral" OR "Neoplasm, Oral" OR "Oral Neoplasm" OR "Oral Neoplasms" OR "Neoplasms, Mouth" OR "Cancer of Mouth" OR "Mouth Cancers" OR "Oral Cancer" OR "Cancer, Oral" OR "Cancers, Oral" OR "Oral Cancers" OR "Cancer of the Mouth" OR "Mouth Cancer" OR "Cancer, Mouth" OR "Cancers, Mouth" OR "Oropharyngeal Neoplasm" OR "Oropharynx Neoplasms" OR "Oropharynx Neoplasm" OR "Neoplasms, Oropharyngeal" OR "Oropharnyx Cancer" OR "Oropharyngeal Cancer" OR "Cancer, Oropharyngeal" OR "Cancers, Oropharyngeal" OR "Oropharyngeal Cancers" OR "Oropharynx Cancer" OR "Cancer, Oropharynx" OR "Cancers, Oropharynx" OR "Oropharynx Cancers" OR "Cancer of the Oropharynx" OR "Otorhinolaryngologic Neoplasm" OR "Otorhinolaryngological Neoplasms" OR "Otorhinolaryngeal Cancer" OR "Ear nose throat cancer" OR "ENT cancer" OR "ORL cancer" OR "Cervicofacial cancer"

#2 TS="Radiodermatitis" OR "Radiodermatitides" OR "radiation dermatitis" OR "Radiation-Induced Dermatitis" OR "Radiation Induced Dermatitis" OR "Dermatitis, Radiation-Induced" OR "Dermatitides, Radiation-Induced" OR "Dermatitis, Radiation Induced" OR "Radiation-Induced Dermatitides" OR "Radiation Recall Dermatitis" OR "Dermatitides, Radiation Recall" OR "Dermatitis, Radiation Recall" OR "Radiation Recall Dermatitides" OR "Radiation Recall Reaction" OR "Radiation Recall Reactions" OR "Reaction, Radiation Recall" OR "Reactions, Radiation Recall" OR "Recall Reaction, Radiation" OR "Recall Reactions, Radiation" OR "radiation induced skin damage" OR "radiation induced skin reaction*" OR RISR OR ARD OR "radiation induced skin injur*" OR "radiation induced skin toxicit*" OR "radiation induced dermatitis" OR "radiation injurie" OR "radiation reaction"

#3 #1 AND #2

**7 Cochrane Library 873**

#1 MeSH descriptor: [Head and Neck Neoplasms] explode all trees

#2 MeSH descriptor: [Nasopharyngeal Carcinoma] explode all trees

#3 MeSH descriptor: [Laryngeal Neoplasms] explode all trees

#4 MeSH descriptor: [Mouth Neoplasms] explode all trees

#5 MeSH descriptor: [Oropharyngeal Neoplasms] explode all trees

#6 MeSH descriptor: [Otorhinolaryngologic Neoplasms] explode all trees

#7 #1 OR #2 OR #3 OR #4 OR #5 OR #6

#8 (Neoplasms, Head and Neck):ab,ti,kw OR (Head, Neck Neoplasms):ab,ti,kw OR (Head and Neck Neoplasm):ab,ti,kw OR (Cancer of Head and Neck):ab,ti,kw OR (Head and Neck Cancer):ab,ti,kw OR (Cancer of the Head and Neck):ab,ti,kw OR (Upper Aerodigestive Tract Neoplasms):ab,ti,kw OR (Neoplasms, UADT):ab,ti,kw OR (UADT Neoplasms):ab,ti,kw OR (Upper Aerodigestive Tract Neoplasm):ab,ti,kw OR (Head Neoplasms):ab,ti,kw OR (Neoplasms, Head):ab,ti,kw OR (Head Neoplasm):ab,ti,kw OR (Neoplasm, Head):ab,ti,kw OR (Neck Neoplasms):ab,ti,kw OR (Neoplasms, Neck):ab,ti,kw OR (Neck Neoplasm):ab,ti,kw OR (Neoplasm, Neck):ab,ti,kw OR (Cancer of Head):ab,ti,kw OR (Head Cancers):ab,ti,kw OR (Head Cancer):ab,ti,kw OR (Cancer, Head):ab,ti,kw OR (Cancers, Head):ab,ti,kw OR (Cancer of the Head):ab,ti,kw OR (Cancer of Neck):ab,ti,kw OR (Neck Cancers):ab,ti,kw OR (Neck Cancer):ab,ti,kw OR (Cancer, Neck):ab,ti,kw OR (Cancers, Neck):ab,ti,kw OR (Cancer of the Neck):ab,ti,kw OR (Carcinoma, Nasopharyngeal):ab,ti,kw OR (Carcinomas, Nasopharyngeal):ab,ti,kw OR (Nasopharyngeal Carcinomas):ab,ti,kw OR (Neoplasms, Laryngeal):ab,ti,kw OR (Laryngeal Neoplasm):ab,ti,kw OR (Neoplasm, Laryngeal):ab,ti,kw OR (Larynx Neoplasms):ab,ti,kw OR (Larynx Neoplasm):ab,ti,kw OR (Neoplasms, Larynx):ab,ti,kw OR (Cancer of Larynx):ab,ti,kw OR (Larynx Cancers):ab,ti,kw OR (Laryngeal Cancer):ab,ti,kw OR (Cancer, Laryngeal):ab,ti,kw OR (Cancers, Laryngeal):ab,ti,kw OR (Laryngeal Cancers):ab,ti,kw OR (Larynx Cancer):ab,ti,kw OR (Cancer, Larynx):ab,ti,kw OR (Cancers, Larynx):ab,ti,kw OR (Cancer of the Larynx):ab,ti,kw OR (Mouth Neoplasm):ab,ti,kw OR (Neoplasm, Mouth):ab,ti,kw OR (Neoplasms, Oral):ab,ti,kw OR (Neoplasm, Oral):ab,ti,kw OR (Oral Neoplasm):ab,ti,kw OR (Oral Neoplasms):ab,ti,kw OR (Neoplasms, Mouth):ab,ti,kw OR (Cancer of Mouth):ab,ti,kw OR (Mouth Cancers):ab,ti,kw OR (Oral Cancer):ab,ti,kw OR (Cancer, Oral):ab,ti,kw OR (Cancers, Oral):ab,ti,kw OR (Oral Cancers):ab,ti,kw OR (Cancer of the Mouth):ab,ti,kw OR (Mouth Cancer):ab,ti,kw OR (Cancer, Mouth):ab,ti,kw OR (Cancers, Mouth):ab,ti,kw OR (Oropharyngeal Neoplasm):ab,ti,kw OR (Oropharynx Neoplasms):ab,ti,kw OR (Oropharynx Neoplasm):ab,ti,kw OR (Neoplasms, Oropharyngeal):ab,ti,kw OR (Oropharnyx Cancer):ab,ti,kw OR (Oropharyngeal Cancer):ab,ti,kw OR (Cancer, Oropharyngeal):ab,ti,kw OR (Cancers, Oropharyngeal):ab,ti,kw OR (Oropharyngeal Cancers):ab,ti,kw OR (Oropharynx Cancer):ab,ti,kw OR (Cancer, Oropharynx):ab,ti,kw OR (Cancers, Oropharynx):ab,ti,kw OR (Oropharynx Cancers):ab,ti,kw OR (Cancer of the Oropharynx):ab,ti,kw OR (Otorhinolaryngologic Neoplasm):ab,ti,kw OR (Otorhinolaryngological Neoplasms):ab,ti,kw OR (Otorhinolaryngeal Cancer):ab,ti,kw OR (Ear nose throat cancer):ab,ti,kw OR (ENT cancer):ab,ti,kw OR (ORL cancer):ab,ti,kw OR (Cervicofacial cancer):ab,ti,kw

#9 #7 OR #8

#10 MeSH descriptor: [Radiodermatitis] explode all trees

#11 (Radiodermatitis):ab,ti,kw OR (Radiodermatitides):ab,ti,kw OR (radiation dermatitis):ab,ti,kw OR (Radiation-Induced Dermatitis):ab,ti,kw OR (Radiation Induced Dermatitis):ab,ti,kw OR (Dermatitis, Radiation-Induced):ab,ti,kw OR (Dermatitides, Radiation-Induced):ab,ti,kw OR (Dermatitis, Radiation Induced):ab,ti,kw OR (Radiation-Induced Dermatitides):ab,ti,kw OR (Radiation Recall Dermatitis):ab,ti,kw OR (Dermatitides, Radiation Recall):ab,ti,kw OR (Dermatitis, Radiation Recall):ab,ti,kw OR (Radiation Recall Dermatitides):ab,ti,kw OR (Radiation Recall Reaction):ab,ti,kw OR (Radiation Recall Reactions):ab,ti,kw OR (Reaction, Radiation Recall):ab,ti,kw OR (Reactions, Radiation Recall):ab,ti,kw OR (Recall Reaction, Radiation):ab,ti,kw OR (Recall Reactions, Radiation):ab,ti,kw OR (radiation induced skin damage):ab,ti,kw OR (radiation induced skin reaction*):ab,ti,kw OR (RISR):ab,ti,kw OR (ARD):ab,ti,kw OR (radiation induced skin injur*):ab,ti,kw OR (radiation induced skin toxicit*):ab,ti,kw OR (radiation induced dermatitis):ab,ti,kw OR (radiation injurie):ab,ti,kw OR (radiation reaction):ab,ti,kw

#12 #10 OR #11

#13 #9 AND #12

**8 EMBASE 1344**

#1 'head and neck tumor'/exp OR 'head and neck cancer'/exp OR 'nasopharynx carcinoma'/exp OR 'larynx tumor'/exp OR 'mouth tumor'/exp OR 'oropharynx tumor'/exp OR 'ear nose throat tumor'/exp

#2 'neoplasms, head and neck':ab,ti OR 'head, neck neoplasms':ab,ti OR 'head and neck neoplasm':ab,ti OR 'cancer of head and neck':ab,ti OR 'head and neck cancer':ab,ti OR 'cancer of the head and neck':ab,ti OR 'upper aerodigestive tract neoplasms':ab,ti OR 'neoplasms, uadt':ab,ti OR 'uadt neoplasms':ab,ti OR 'upper aerodigestive tract neoplasm':ab,ti OR 'head neoplasms':ab,ti OR 'neoplasms, head':ab,ti OR 'head neoplasm':ab,ti OR 'neoplasm, head':ab,ti OR 'neck neoplasms':ab,ti OR 'neoplasms, neck':ab,ti OR 'neck neoplasm':ab,ti OR 'neoplasm, neck':ab,ti OR 'cancer of head':ab,ti OR 'head cancers':ab,ti OR 'head cancer':ab,ti OR 'cancer, head':ab,ti OR 'cancers, head':ab,ti OR 'cancer of the head':ab,ti OR 'cancer of neck':ab,ti OR 'neck cancers':ab,ti OR 'neck cancer':ab,ti OR 'cancer, neck':ab,ti OR 'cancers, neck':ab,ti OR 'cancer of the neck':ab,ti OR 'carcinoma, nasopharyngeal':ab,ti OR 'carcinomas, nasopharyngeal':ab,ti OR 'nasopharyngeal carcinomas':ab,ti OR 'neoplasms, laryngeal':ab,ti OR 'laryngeal neoplasm':ab,ti OR 'neoplasm, laryngeal':ab,ti OR 'larynx neoplasms':ab,ti OR 'larynx neoplasm':ab,ti OR 'neoplasms, larynx':ab,ti OR 'cancer of larynx':ab,ti OR 'larynx cancers':ab,ti OR 'laryngeal cancer':ab,ti OR 'cancer, laryngeal':ab,ti OR 'cancers, laryngeal':ab,ti OR 'laryngeal cancers':ab,ti OR 'larynx cancer':ab,ti OR 'cancer, larynx':ab,ti OR 'cancers, larynx':ab,ti OR 'cancer of the larynx':ab,ti OR 'mouth neoplasm':ab,ti OR 'neoplasm, mouth':ab,ti OR 'neoplasms, oral':ab,ti OR 'neoplasm, oral':ab,ti OR 'oral neoplasm':ab,ti OR 'oral neoplasms':ab,ti OR 'neoplasms, mouth':ab,ti OR 'cancer of mouth':ab,ti OR 'mouth cancers':ab,ti OR 'oral cancer':ab,ti OR 'cancer, oral':ab,ti OR 'cancers, oral':ab,ti OR 'oral cancers':ab,ti OR 'cancer of the mouth':ab,ti OR 'mouth cancer':ab,ti OR 'cancer, mouth':ab,ti OR 'cancers, mouth':ab,ti OR 'oropharyngeal neoplasm':ab,ti OR 'oropharynx neoplasms':ab,ti OR 'oropharynx neoplasm':ab,ti OR 'neoplasms, oropharyngeal':ab,ti OR 'oropharnyx cancer':ab,ti OR 'oropharyngeal cancer':ab,ti OR 'cancer, oropharyngeal':ab,ti OR 'cancers, oropharyngeal':ab,ti OR 'oropharyngeal cancers':ab,ti OR 'oropharynx cancer':ab,ti OR 'cancer, oropharynx':ab,ti OR 'cancers, oropharynx':ab,ti OR 'oropharynx cancers':ab,ti OR 'cancer of the oropharynx':ab,ti OR 'otorhinolaryngologic neoplasm':ab,ti OR 'otorhinolaryngological neoplasms':ab,ti OR 'otorhinolaryngeal cancer':ab,ti OR 'ear nose throat cancer':ab,ti OR 'ent cancer':ab,ti OR 'orl cancer':ab,ti OR 'cervicofacial cancer':ab,ti OR 'head and neck neoplasms':ab,ti OR 'laryngeal neoplasms':ab,ti OR 'mouth neoplasms':ab,ti OR 'oropharyngeal neoplasms':ab,ti OR 'otorhinolaryngologic neoplasms':ab,ti

#3 #1 OR #2

#4 'radiation dermatitis'/exp OR 'radiation dermatitis'

#5 'radiodermatitis':ab,ti OR 'radiodermatitides':ab,ti OR 'radiation-induced dermatitis':ab,ti OR 'dermatitis, radiation-induced':ab,ti OR 'dermatitides, radiation-induced':ab,ti OR 'dermatitis, radiation induced':ab,ti OR 'radiation-induced dermatitides':ab,ti OR 'radiation recall dermatitis':ab,ti OR 'dermatitides, radiation recall':ab,ti OR 'dermatitis, radiation recall':ab,ti OR 'radiation recall dermatitides':ab,ti OR 'radiation recall reaction':ab,ti OR 'radiation recall reactions':ab,ti OR 'reaction, radiation recall':ab,ti OR 'reactions, radiation recall':ab,ti OR 'recall reaction, radiation':ab,ti OR 'recall reactions, radiation':ab,ti OR 'radiation induced skin damage':ab,ti OR 'radiation induced skin reaction*':ab,ti OR 'risr':ab,ti OR 'ard':ab,ti OR 'radiation induced skin injur*':ab,ti OR 'radiation induced skin toxicit*':ab,ti OR 'radiation induced dermatitis':ab,ti OR 'radiation injurie':ab,ti OR 'radiation reaction':ab,ti

#6 #4 OR #5

#7 #3 AND #6

**9 CINAHL 101**

#1 MH "Head and Neck Neoplasms"

#2 SU "Nasopharyngeal Carcinoma" OR "Laryngeal Neoplasms" OR "Mouth Neoplasms" OR "Oropharyngeal Neoplasms" OR "Otorhinolaryngologic Neoplasms" OR "Neoplasms, Head and Neck" OR "Head, Neck Neoplasms" OR "Head and Neck Neoplasm" OR "Cancer of Head and Neck" OR "Head and Neck Cancer" OR "Cancer of the Head and Neck" OR "Upper Aerodigestive Tract Neoplasms" OR "Neoplasms, UADT" OR "UADT Neoplasms" OR "Upper Aerodigestive Tract Neoplasm" OR "Head Neoplasms" OR "Neoplasms, Head" OR "Head Neoplasm" OR "Neoplasm, Head" OR "Neck Neoplasms" OR "Neoplasms, Neck" OR "Neck Neoplasm" OR "Neoplasm, Neck" OR "Cancer of Head" OR "Head Cancers" OR "Head Cancer" OR "Cancer, Head" OR "Cancers, Head" OR "Cancer of the Head" OR "Cancer of Neck" OR "Neck Cancers" OR "Neck Cancer" OR "Cancer, Neck" OR "Cancers, Neck" OR "Cancer of the Neck" OR "Carcinoma, Nasopharyngeal" OR "Carcinomas, Nasopharyngeal" OR "Nasopharyngeal Carcinomas" OR "Neoplasms, Laryngeal" OR "Laryngeal Neoplasm" OR "Neoplasm, Laryngeal" OR "Larynx Neoplasms" OR "Larynx Neoplasm" OR "Neoplasms, Larynx" OR "Cancer of Larynx" OR "Larynx Cancers" OR "Laryngeal Cancer" OR "Cancer, Laryngeal" OR "Cancers, Laryngeal" OR "Laryngeal Cancers" OR "Larynx Cancer" OR "Cancer, Larynx" OR "Cancers, Larynx" OR "Cancer of the Larynx" OR "Mouth Neoplasm" OR "Neoplasm, Mouth" OR "Neoplasms, Oral" OR "Neoplasm, Oral" OR "Oral Neoplasm" OR "Oral Neoplasms" OR "Neoplasms, Mouth" OR "Cancer of Mouth" OR "Mouth Cancers" OR "Oral Cancer" OR "Cancer, Oral" OR "Cancers, Oral" OR "Oral Cancers" OR "Cancer of the Mouth" OR "Mouth Cancer" OR "Cancer, Mouth" OR "Cancers, Mouth" OR "Oropharyngeal Neoplasm" OR "Oropharynx Neoplasms" OR "Oropharynx Neoplasm" OR "Neoplasms, Oropharyngeal" OR "Oropharnyx Cancer" OR "Oropharyngeal Cancer" OR "Cancer, Oropharyngeal" OR "Cancers, Oropharyngeal" OR "Oropharyngeal Cancers" OR "Oropharynx Cancer" OR "Cancer, Oropharynx" OR "Cancers, Oropharynx" OR "Oropharynx Cancers" OR "Cancer of the Oropharynx" OR "Otorhinolaryngologic Neoplasm" OR "Otorhinolaryngological Neoplasms" OR "Otorhinolaryngeal Cancer" OR "Ear nose throat cancer" OR "ENT cancer" OR "ORL cancer" OR "Cervicofacial cancer"

#3 #1 OR #2

#4 MH "Radiodermatitis"

#5 SU "Radiodermatitis" OR "Radiodermatitides" OR "radiation dermatitis" OR "Radiation-Induced Dermatitis" OR "Radiation Induced Dermatitis" OR "Dermatitis, Radiation-Induced" OR "Dermatitides, Radiation-Induced" OR "Dermatitis, Radiation Induced" OR "Radiation-Induced Dermatitides" OR "Radiation Recall Dermatitis" OR "Dermatitides, Radiation Recall" OR "Dermatitis, Radiation Recall" OR "Radiation Recall Dermatitides" OR "Radiation Recall Reaction" OR "Radiation Recall Reactions" OR "Reaction, Radiation Recall" OR "Reactions, Radiation Recall" OR "Recall Reaction, Radiation" OR "Recall Reactions, Radiation" OR "radiation induced skin damage" OR "radiation induced skin reaction*" OR RISR OR ARD OR "radiation induced skin injur*" OR "radiation induced skin toxicit*" OR "radiation induced dermatitis" OR "radiation injurie" OR "radiation reaction"

#6 #4 OR #5

#7 #3 AND #6

**10 JBI EBP Database 4**

#1 ("Radiodermatitis" or "Radiodermatitides" or "radiation dermatitis" or "Radiation-Induced Dermatitis" or "Radiation Induced Dermatitis" or "Dermatitis, Radiation-Induced" or "Dermatitides, Radiation-Induced" or "Dermatitis, Radiation Induced" or "Radiation-Induced Dermatitides" or "Radiation Recall Dermatitis" or "Dermatitides, Radiation Recall" or "Dermatitis, Radiation Recall" or "Radiation Recall Dermatitides" or "Radiation Recall Reaction" or "Radiation Recall Reactions" or "Reaction, Radiation Recall" or "Reactions, Radiation Recall" or "Recall Reaction, Radiation" or "Recall Reactions, Radiation" or "radiation induced skin damage" or "radiation induced skin reaction*" or RISR or ARD or "radiation induced skin injur*" or "radiation induced skin toxicit*" or "radiation induced dermatitis" or "radiation injurie" or "radiation reaction").ab,kw,ti.

**11 Up To Date 2**

**12 Other Guideline Websites and Professional Associations 2**
